# Development of a Social Play Evaluation Tool for Preschool Children

**DOI:** 10.3390/healthcare10010102

**Published:** 2022-01-05

**Authors:** Sun-Hee Lee, Ickpyo Hong, Hae Yean Park

**Affiliations:** 1 Rodem Pediatric Clinic Child Development Clinic, Seoul 07631, Korea; belleesunny@naver.com; 2 Department of Occupational Therapy, College of Software and Digital Healthcare Convergence, Yonsei University, Wonju 26493, Korea; ihong@yonsei.ac.kr

**Keywords:** social play, Rasch analysis, development of evaluation tool, preschool children

## Abstract

Play has been used as an intervention or evaluation tool for children. Developing a social play evaluation tool can provide clinical criteria for intervening in social play. We aimed to develop a social play evaluation tool for preschool children based on Parten’s stages of development. We tested the construct validity of the scale using confirmatory factor analysis and Rasch analysis, and the known-groups validity by examining the Rasch-calibrated mean score differences across age groups. A total of 40 preliminary items—17 items for associative play and 23 items for cooperative play—were examined. There were significant differences in the scores for associative play between ages 3 and 6 (*F* = 2.65, *p* = 0.049), and for cooperative play between ages 3 and 5, 3 and 6, and 4 and 6 (*F* = 10.44, *p* < 0.0001). The findings could contribute to subsequent development and validation of occupational therapy programs on play.

## 1. Introduction

Since the early days of occupational therapy, play has been included and used as a sleep are the four rhythms that comprise a human life [1]. In an early study on play, Alessandrini (1949) [2] mentioned that play is an important activity that must not be mistaken as being a waste of time. The theoretical framework for occupational behavior proposed by Reilly (1974) [3] directed attention to play in occupational therapy and play research, and confirmed the importance of play as a therapeutic means.

Play has also been described as a platform for developing sensory integration, physical abilities, cognitive and language skills, and interpersonal relationships [4]. Moreover, play is a medium through which young and older children can move their bodies, engage in relationships with other people, solve problems, and learn how to cope with their emotions, as well as stimulating the growth of various areas, further highlighting the importance for children to participate in play [5]. It is critical to measure play, as it is a child’s primary occupation and thus, provides valuable information about the child’s abilities and functions [6,7]. In addition, given the weight of play in children’s lives, occupational therapists can use it as a means to evaluate and treat them [8].

A study on the trends of the use of evaluation tools among pediatric occupational therapists showed that the Revised Knox Preschool Play Scale and the Test of Playfulness are the most commonly utilized tools [9]. Both these scales assess preschool children’s play by directly observing play in a child’s familiar environment [4,7]. However, the utilization of these observational tools can be challenging for busy clinicians. For instance, clinicians should spend about 60 min to complete the Revised Knox Preschool Play Scale. In addition, as these tools are designed to be used outside of clinical settings, it often contributes to their low use by occupational therapists [10].

Further, a survey on the perception of play among occupational therapists confirmed that these professionals generally evaluate play through interviews and clinical observations [11]. Nevertheless, in a survey on pediatric occupational therapists regarding evaluation areas that need to be utilized in occupational therapy settings, many of the participants pinpointed play [9]. In other words, despite the perceived importance of play evaluation by occupational therapists, official play evaluation is rarely performed. In fact, occupational therapists in practice have reported that barriers such as lack of training on play evaluation, lack of time, problems with role boundaries, and funding matters can hinder play evaluation in clinical settings [12,13]. This situation is also common in Korea, where occupational therapists have reported their wish to utilize play in occupational therapy, but are in need of play spaces, tools, evaluation tools, and organizational support, as well training or refresher courses on play [11].

In fact, most therapists do not aim for play or focus on play as a result of their intervention. If play is undervalued as an end in itself, it should be replaced by a more measurable alternative [14]. Although occupational therapists are not the only professions related to the multifaceted phenomenon of play, occupational therapy has a unique perspective on the meaning and importance of play itself as an occupation [12].

Evaluating peer social interaction and social play among children is a complicated process, and because these two constructs cannot be directly tested, they must be evaluated through natural observation in social situations, or by a person who frequently observes the child [15]. The existing peer play behavior scale for children, validated by Choi and Shin (2008) was based on the Penn Interactive Peer Play Scale [16]; however, it has been developed for use by teachers, rather than by parents. A parent-reported questionnaire has many advantages, such as the fact that it is easy for an expert to evaluate a child directly or to obtain additional information on child development for diagnosis, reflecting the behavior from various environments outside the treatment room, and including the parent directly in the evaluation and intervention process [17,18]. Choi (2010) also reported that there is a need for evaluation tools that can be appropriately used by parents, which supports the rationale for this study in developing an evaluation tool that can be used by parents [19].

Parten classified children’s social play into unoccupied behavior, onlooker behavior, solitary play, parallel play, associative play, and cooperative play [20,21]. Among them, associative play and cooperative play involves more complex interactions with peers, require a higher level of participation, and the ability to initiate, maintain, and terminate activities in a socially appropriate manner [22,23,24].

A close look at children’s social play shows that it can be divided into two types, including playing alone and playing with peers. Once children reach the age of 3 or 4, solitary play and parallel play begin to decline, while associative play and cooperative play begin to increase as they come into contact with their peers more frequently [20]. If a child aged 4 or 5 years old rarely participates in group play with peers (associative play and cooperative play) and primarily engages in solitary play or parallel play, it is necessary to observe the child’s play more closely [25].

Children’s social play increases in quantity with advancing age, and children with highly social skills frequently engage in social play, such as appropriately attempting and maintaining interaction with peers. However, children with low social skills tend to participate in social play with peers less frequently, which can decrease the opportunity to develop their social skills. Social play helps children develop social skills, and social skills, in turn, it can influence a child’s social play with their peers; thus, children’s social play and social skills complement each other [26]. Kindergarten-aged children gradually increase in-group play, such as associative play and cooperative play [27,28,29]. Therefore, associative play and cooperative play were organized into sub-areas. Based on a previous study on preschool children in Korea, this study was conducted with 3–6 years old [30,31].

This study aims to develop a social play evaluation tool for preschool children by testing the validity of the items developed using the Rasch model; and to assess whether these items could be used by professionals in the clinical field.

## 2. Methods

### 2.1. Design

From a previous Delphi study, a total of 40 items were developed, with 17 items for associative play and 23 items for cooperative play [32]. This instrument consists of a four-point Likert scale (Never—1, Rarely—2, Sometimes—3, Always—4). Parents of preschool children completed the test items. The construct validity (confirmatory factor analysis, Rasch analysis) and Known-groups validity were tested using the test items obtained from the previous Delphi study [32]. This study that does not meet the criteria for human subjects’ research was exempted by the institutional review board at Yonsei university [YUWIRB-1041849-202004-BM-041-03].

### 2.2. Participants

The study was conducted nationwide on parents of preschool children aged 3–6 years in South Korea. A total of 310 children who do not have a medical problem were enrolled. Among the participants, parents were 36 males (11.6%) and 274 females (88.4%). Among the participants, there were 129 (41.6%) boys and 181 (58.4%) girls. Regarding age, there were 78 (25.2%) 3-year-olds, 98 (31.6%) 4-year-olds, 78 (25.2%) 5-year-olds, and 56 (18.1%) 6-year-olds. Education facilities included home (*n* = 5, 1.6%), daycare center (*n* = 152, 49.0%), kindergarten (*n* = 144, 46.5%), play group (*n* = 5, 1.6%), and English kindergarten (*n* = 4, 1.4%).

### 2.3. Procedures

We recruited the participants in person by visiting daycare centers and kindergartens, and asked the participants to complete the evaluation form. The evaluation form was distributed directly or via mail, and retrieved the same way. Further, an online survey using a Google survey was also performed concurrently.

The content of each item on the evaluation form was read and parents marked the column that most closely corresponds to the child’s behavior and reaction. Based on the four-point Likert scale, scores were derived for 17 items of associative play and 23 items of cooperative play. Detailed information on the evaluation tools and recruitment of this study can be found in previous studies [32].

A total of 350 forms were distributed, 323 of which were retrieved between August 2019 and September 2019. After excluding 13 forms for inappropriate age, or careless, missing, or inaccurate responses, a total of 310 forms were included in the final analysis. The final sample size was sufficient for confirmatory factor analysis and Rasch analysis [33,34].

### 2.4. Data Analysis

We tested the construct validity using confirmatory factor analysis (CFA) and Rasch analysis and examined known-groups validity. One of the core assumptions in Rasch analysis is the unidimensional assumption [35]. We conducted CFA to check if there was dominant measurement structure(s) in the test items. The factor structure(s) of the test items was tested using a CFA with a two-factor model. Model fit was assessed using the following fit indices, including CMIN/DF(χ2/df), root mean square error of approximation (RMSEA), comparative fit index (CFI), and Tucker–Lewis index (TLI). The cutoffs for the indices were set to ≤3.0 for CMIN/DF, ≤0.08 for RMSEA, and ≥0.90 for CFI and TLI [36,37].

Rasch analysis was performed using a rating scale model. We analyzed item fit, person fit, item difficulty hierarchy, rating scale structure, and precision (person strata). In this study, the criteria for item and person misfit were set to infit or outfit mean square (MNSQ) of <0.6 or >1.4 with a z-standardized (ZSTD) value of <−2.0 or >2.0 [38,39]. For item difficulty, the differences in skills for the item were compared using a logit score, which is computed by converting the raw score obtained from an ordinal scale (Likert scale) into an interval scale, and by examining the order and interval of the difficulties of the item on a straight line [40,41]. For the rating scale analysis, the appropriate rating scale structure was set to: (1) a category count of ≥10, (2) vertical hierarchy, where average measure increases with an increased rating (monotonicity), and (3) outfit MNSQ < 2.0.

For the precision test, we considered greater than 3.0 person strata as “excellent” precision that is equivalent to a traditional reliability value of 0.90 [40]. The person separation index (G) was calculated as the number of statistically distinct person strata identified by the following formula [41].

Number of Person strata = (4 × G + 1)/3

Finally, known-groups validity was tested based on the differences in the mean logit scores by age, followed by the Tukey test as a post-hoc analysis when ANOVA was performed. The descriptive statistics was conducted using the SPSS version 18.0. CFA was performed using Mplus version 8.0. Rasch analysis was performed using the Winstep version 3.92.1 (Chicago, IL, USA). Inferential tests were performed using the using SAS version 9.4 (SAS Institute, Cary, NC, USA).

## 3. Results

### 3.1. Step 1. CFA

CFA was performed to test the fit of a model comprising two areas, with 17 items for associative play and 23 items for cooperative play, on 310 children aged 3–6 years. The results confirmed a good fit, with the following fit indices: CMIN/DF = 2.651, RMSEA = 0.073, SRMR = 0.070, CFI = 0.914, and TLI = 0.909. The factor correlation between the two factors (associative play area and cooperative play area) was 0.85. Based on the identified factor structures, we conducted individual Rasch analysis for each play area (associative play area and cooperative play).

### 3.2. Step 2. Rasch Analysis

#### 3.2.1. Item and Person Fit

From the 17 items for associative play, three items misfit the Rasch model, including item 10 “The child is more focused on his/her own interests rather than on the group (e.g., The child is very interested in playing along with friends but wants to play as he/she wants.)” (MNSQ = 1.88, ZSTD = 8.64); item 14 “The child acts on his/her own will and does not subordinate his/her interests to the group’s demands.” (MNSQ = 1.46, ZSTD = 5.33); and item 16 “The child plays with another child without distinguishing roles.” (MNSQ = 1.41, ZSTD = 3.91). One out of 23 items for cooperative play misfit the Rasch model, item 33 “The child has his/her own ranking of children that he/she likes and behaves differently according to which other child(ren) is involved in play” (MNSQ = 1.82, ZSTD = 7.98) (Table 1). Misfitting participants (*n* = 75) were excluded, including 30 out of 310 (9.6%) participants in the associative play and 45 out of 310 (14.5%) participants in the cooperative play.

#### 3.2.2. Item Difficulty and Person-Item Match

**Figure 1:**
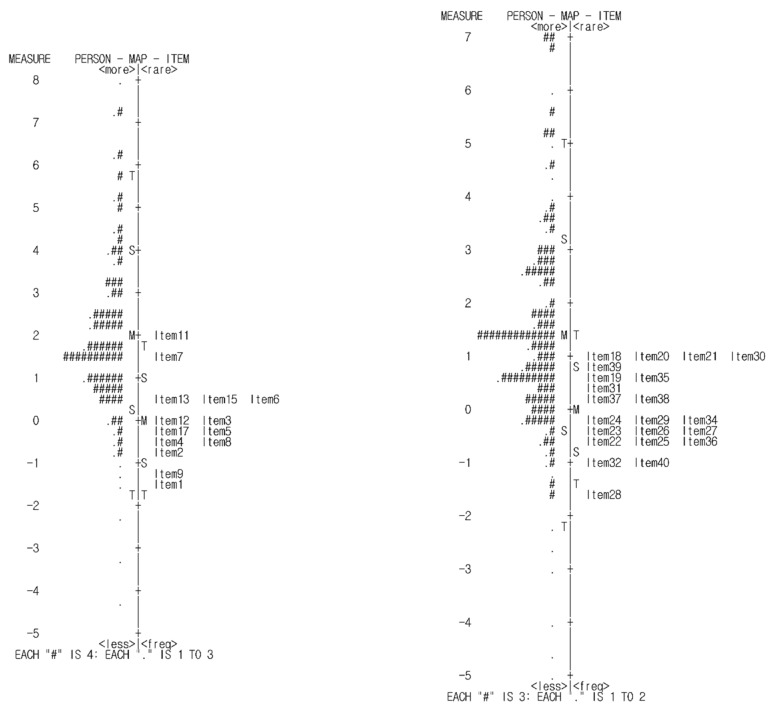
Graphic presentation of the relationship among person ability and item difficulty for the associative play area (**left**) and cooperative play area (**right**).

After excluding misfitting items and participants, the remaining items were re-calibrated in a hierarchy of difficulty (Figure 1). In this study, the difficulty range of the items and the distribution of participants were appropriately achieved. In addition, the differences in the difficulty levels of each item were shown, indicating sensitivity to changes during measurement. Among the 14 items for associative play, the most difficult item was “The child kind of tries to control another child with whom he/she”,(1.75 logits) followed by “The child tries to recruit another child who would participate in the group” (1.33 logits). The easiest item was “The child prefers to play with other children in a group” (−1.38 logits) followed by “The child plays by interacting with another child, such as by asking questions” (−1.02 logits). Among the 22 items for cooperative play, the most difficult item was “The child organizes play, such as assigning work to different people within a group” (1.07 logits) and the easiest item was “The child engages in role play with other children by creating various scenarios (example: hospital, teacher)” (−1.64 logits).

#### 3.2.3. Rating Scale Analysis

**Figure 2:**
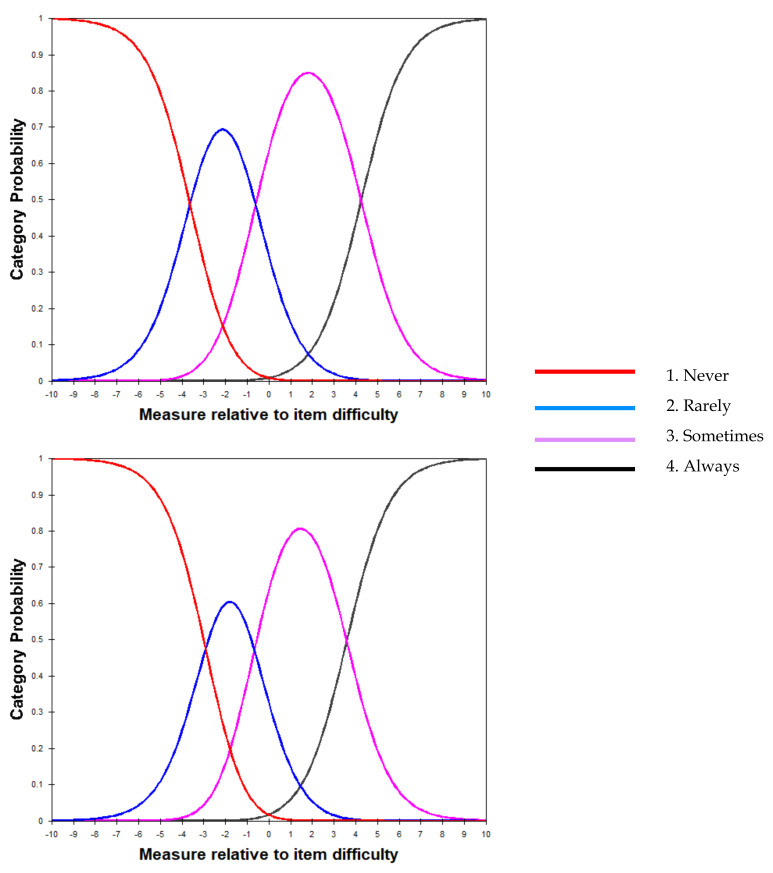
Graphic presentation of the probability of responses for each of the four ratings (top: associative play area; bottom: cooperative play area).

Regarding the fit of the four-point rating scale (1, 2, 3, 4), the category count was at least 10 or greater in both associative play and cooperative play areas, with a vertical hierarchy, where the average measure increases with increasing rating, and the outfit MNSQ value was smaller than 2.0, confirming that the rating scale is appropriate (Table 2, Figure 2).

#### 3.2.4. Precision

In the associative play area, the separation index was 2.89, person strata was 4.18 and the separation reliability was 0.89. In the cooperative play area, the separation index was 4.28, the person strata was 6.04 and the separation reliability was 0.95.

### 3.3. Step 3. Known-Groups Validity

In terms of the differences in the Rasch-calibrated mean scores by age in the associative play area (*F* = 2.65, *p* = 0.0492), there were significant differences between ages 3 and 6 (mean difference = 0.97 logits, *p* < 0.05). In the cooperative play area, there were significant differences (*F* = 10.44, *p* < 0.0001) between ages 3 and 5 (mean difference = 1.43 logits, *p* < 0.05), ages 3 and 6 (mean difference = 2.32 logits, *p* < 0.05), and ages 4 and 6 (mean difference = 1.83 logits, *p* < 0.05).

## 4. Discussion

In this study, we examined the construct validity of the Yonsei-Social Play Evaluation Tool (Y-SPET). The CFA with a two-factor model was performed to test the fit of 40 items, which consisted of 17 items for associative play and 23 items for cooperative play. All CFA fit indices for the factor structure (associative play and cooperative play) of the evaluation tool met the cutoff, which confirms that the factor structure is suitable for individual Rasch analysis.

Rasch analysis results showed that three misfitting items in the associative play area. Of these, two of these items seem to have had poor fit, as they describe parallel play, a limited form of social participation, as opposed to associative play, where children communicate and interact with their peers. The misfitting item in the cooperative play area is presumably influenced by a stereotype suggestive of negative social play attitudes.

For person fit, an infit Z value of greater than 2.0 signifies that the evaluator applied overly strict criteria, and a value of below −2.0 suggests lenient scoring [42]. Moreover, the person fit is deemed inappropriate when the participant gives an inappropriate response by guessing or making a mistake, when the participant is anxious during testing or faces technical problems, when there are problems due to a lack of experience, and when there are problems with learning specific contents in the items or the item is too easy [43]. With the exception of 30 participants with inappropriate person fit for associative play, and 1 participant out of 45 with inappropriate person fit for cooperative play, all participants had an infit Z value of greater than 2.0, suggesting that the evaluators (i.e., the parents) applied strict scoring criteria when evaluating their children [42].

The most difficult items in the associative play area were “The child kind of tries to control another child with whom he/she wants or does not want to play” and “The child tries to recruit another child who would participate in the group”. This seems to be attributable to the need to interpret the terms “control,” which signifies that the child tries to manipulate or control the situation, and “recruit”, which means that the child tries to bring another child into crossing a particular boundary. The easiest item in the associative play area was “The child prefers to play with other children in a group” and “The child plays by interacting with another child, such as by asking questions”. These describe typical child behaviors, where a child wishes to play with another child. In the cooperative play area, the most difficult item was “The child organizes play, such as assigning work to different people within a group”. This seems to be attributable to the fact that this item describes an advanced level of play behavior, such as establishing a system or order within a group. The easiest item was “The child engages in role play with other children, creating various scenarios (e.g., hospital, teacher)”. This item describes cooperative play that includes role allocation and play theme setting with a shared goal in a group.

Regarding the rating scale, a four-point scale was found to be appropriate for both associative play and cooperative play areas, as confirmed by the even distribution of the probability curves of the rating scale, which confirms that the scale categories are independent [44]. This supports previous findings that a four-point rating scale is appropriate based on the children’s peer play behavior scale [45]. and the Korean version of the Social Skill Rating System for preschool level [46].

A separation reliability closer to 1.0 indicates higher consistency [47]. In this study, the person separation reliability and item separation reliability were 0.89 and 0.98, respectively, for associative play, and 0.95 and 0.97, respectively, for cooperative play, confirming high consistency. This suggests that the items in the evaluation tool developed in this study are clearly independent and differentiate varying degrees of social play in the participants.

The final version of the social play evaluation tool for preschool children developed in this study had a total of 36 items, with 14 items for associative play and 22 items for cooperative play in Appendix A. This tool addresses the limitations of the Preschool Play Behavior Scale (PPBS) [48]. adapted and modified by Cho (2002), which only consists of 18 items [49]. Furthermore, the assessment tool developed in this study is intended for guardians and parents who have observed their children’s behavior for a long period, which makes them more competent than anyone else in assessing their behavior. In addition, if parents have a good understanding of the assessment questions, and have carefully observed the child’s recent behavior, their reports can be considered reliable. Parental assessments of child and peer interactions can be conducted from various perspectives with the development of appropriate tools [45,50].

Further, in the known-groups validity testing, there were differences in associative play between ages 6 and 3, and in cooperative play between ages 6 and 4, 6 and 3, and 5 and 3. This supports the findings of Parten (1932) that the level of play changes as children age and acquire social skills, showing changes in associative play at ages 3.5–4.5 years, and changes in cooperative play at age 4.5 years.

### 4.1. Limitations

This study has several limitations. First, the evaluation scores of the assessment tool developed in this study are limited in that they provide little specific information for intervention plans. Second, this study did not specifically identify the social play difficulties of children with different types of disabilities. Despite these limitations, however, the social play assessment tool for preschool children developed through this study is significant in that parents are able to understand the levels of social play. Third, the CFA indicated the instrument consists of two latent variables (associative play and cooperative play) and there was a strong correlation between the two latent variables (r = 0.85). While the individual play areas fit to the Rasch model, a bifactor model could demonstrate a better model fit with the study data (e.g., general factor (social play) and specific factors (associative play and cooperative play)). Lastly, while the instrument was calibrated by the Rasch model, conventional psychometric testing (e.g., test–retest reliability, convergent or divergent validity) was conducted.

### 4.2. Future Lines of Research

The study findings suggested that a shortened version of the evaluation tool can be developed for the quick evaluation of children within a set time. In addition, the results of the evaluation should be included in children’s records, to inform the planning of interventions by occupational therapists. A study that applied these practices in the clinical field should be done.

## 5. Conclusions

The development of a social play evaluation tool for preschool children in this study is significant in that it presents evidence for intervening in social play. The evaluation tool could contribute to development and assessment of the effects of programs for social play in preschool children in the field of occupational therapy.

## Figures and Tables

**Table 1:** Item fit statistics on associative play and cooperative play.

Area	Item	Measure (Logits)	Infit		Outfit	
			MNSQ	ZSTD	MNSQ	ZSTD
Associative play	1	−1.38	0.90	−1.23	0.90	−1.02
	2	−0.69	1.23	2.34	1.27	2.31
	3	0.04	1.27	2.73	1.32	2.91
	4	−0.55	0.81	−2.15	0.75	−2.55
	5	−0.26	0.85	−1.71	0.79	−2.20
	6	0.31	0.94	−0.65	0.92	−0.81
	7	1.33	1.06	0.78	1.12	1.39
	8	−0.29	0.65	−4.23	0.60	−4.48
	9	−1.02	0.65	−4.61	0.58	−4.45
	10	Deleted				
	11	1.75	1.26	3.05	1.34	3.78
	12	−0.13	1.00	0.04	0.95	−0.49
	13	0.53	1.40	4.04	1.38	3.60
	14	Deleted				
	15	0.48	0.89	−1.24	0.90	−1.02
	16	Deleted				
	17	−0.12	0.98	−0.22	0.95	−0.46
Cooperative play	18	0.94	0.92	−0.97	0.94	−0.68
	19	0.67	1.15	1.65	1.16	1.64
	20	1.06	0.88	−1.46	0.90	−1.10
	21	1.07	0.90	−1.26	0.89	−1.19
	22	−0.53	0.68	−3.85	0.63	−3.99
	23	−0.49	0.94	−0.68	0.88	−1.15
	24	−0.11	0.88	−1.35	0.84	−1.70
	25	−0.60	0.68	−3.95	0.62	−4.18
	26	−0.40	1.03	0.39	0.98	−0.15
	27	−0.33	0.91	−0.93	0.89	−1.10
	28	−1.64	1.21	2.38	1.14	1.07
	29	−0.27	1.12	1.29	1.01	0.17
	30	0.97	1.04	0.47	1.10	1.13
	31	0.45	1.10	1.11	1.02	0.29
	32	−0.97	1.05	0.59	0.99	−0.03
	33	Deleted				
	34	−0.13	1.16	1.70	1.18	1.69
	35	0.69	0.85	−1.79	0.88	−1.27
	36	−0.53	1.32	3.21	1.27	2.42
	37	0.19	1.25	2.59	1.35	3.24
	38	0.15	0.85	−1.74	0.89	−1.14
	39	0.77	0.91	−1.02	0.94	−0.60
	40	−0.97	1.05	0.57	0.98	−0.11

MnSq, mean square standardized residual; Z STD, standardized Z value. Acceptable range: MnSq ≥ 0.6 and ≤1.4.

**Table 2:** Results of rating scale category statistics for the Associative play and Cooperative play.

	Category	Obs. Average	Infit MNSQ	Outfit MNSQ	Structure Calibration
Associative play	1	−2.01	1.18	1.30	None
	2	−0.01	1.02	1.00	−3.64
	3	1.80	0.99	0.92	−0.61
	4	4.57	0.97	0.95	4.25
Cooperative play	1	−4.00	0.81	0.77	None
	2	−0.41	1.01	0.98	−4.02
	3	1.73	1.00	0.98	−0.73
	4	5.15	1.01	0.95	4.75

## Data Availability

The data that support the finding of this study are available on request from the corresponding author, [H.Y.P.]. The data are not publicly available due to restrictions.

## Appendix A. Scoring Sheets

**Table A1:** Associative play.

Item	Associative Play	Never	Rarely	Sometimes	Always
1	The child prefers to play with other children in a group				
2	The child plays by interacting with another child, such as by asking questions				
3	The child plays and sing or dances with other children in the group.				
4	The child talk with other children about the story of the play activity				
5	The child plays with other children by lending and borrowing toys				
6	The child share, lend, and order toys while preoccupied with their own play activities				
7	The child plays and exchanges toys with other children, although the story of the play is unstructured				
8	The child tells the parent or caregiver the story of the play with another child				
9	The child engages in the play of other children				
10	The child notices and wants to engage with a child who likes the same style of play				
11	The child pay attention to other children’s play activities and engages in many conversations.				
12	The child can offer his (or her) toy to another child				
13	The child tries to recruit another child who would participate in the group				
14	The child kind of tries to control another child with whom he/she				

**Table A2:** Cooperative play.

Item	Cooperative Play	Never	Rarely	Sometimes	Always
1	The child engages in role play with other children, creating various scenarios (e.g., hospital, teacher)				
2	The child can compose a play environment with other children (e.g., bringing toys, clearing obstacles, etc.)				
3	The child participates in the activity by selecting and sharing roles with other children in “pretend” play situations (e.g., role play)				
4	The child remembers previous play with other children and is able to continue it the next time they meet				
5	The child engage in play activities with rules				
6	The child can play given roles in the process of play with other children				
7	The child can help other children in need during play				
8	The child play with three or more children without constant supervision				
9	The child play cooperatively with other children to complete objects or structures				
10	The child can play to achieve competitive goals				
11	The child explain the rules of play to other children				
12	The child can transform one play into another				
13	When a child is given an unwanted role in play, the child can express his (or her) opinion				
14	The child can change their roles in play for other children				
15	The child understand the rules of fair play				
16	The child compete against other teams in a game with rules				
17	The child can work with other children to create new ways of playing or rules				
18	The child try to conform to their own and other children’s opinions when making play choices				
19	The child form teams to play in groups				
20	The child share roles in play to complete the outcome				
21	The child can set goals for play activities within a group and play proceeds systematically				
22	The child organizes play, such as assigning work to different people within a group				
